# Resistance to first-line antibiotic therapy among patients with uncomplicated acute cystitis in Melbourne, Australia: prevalence, predictors and clinical impact

**DOI:** 10.1093/jacamr/dlad145

**Published:** 2023-12-28

**Authors:** Stephanie J Curtis, Jason C Kwong, Yee Lin Chaung, Danielle Mazza, Calum J Walsh, Kyra Y Chua, Andrew J Stewardson

**Affiliations:** Department of Infectious Diseases, The Alfred Hospital and Central Clinical School, Monash University, 85 Commercial Road, Melbourne, VIC 3004, Australia; Department of Microbiology & Immunology, The University of Melbourne at the Peter Doherty Institute for Infection & Immunity, 792 Elizabeth Street, Melbourne, VIC 3000, Australia; Heathmont General Practice, 220 Canterbury Road, Heathmont, VIC 3135, Australia; Department of General Practice, Monash University, 1/270 Ferntree Gully Road, Notting Hill, VIC 3168, Australia; Doherty Applied Microbial Genomics, Department of Microbiology & Immunology, The University of Melbourne at the Peter Doherty Institute for Infection & Immunity, 792 Elizabeth Street, Melbourne VIC 3000, Australia; Department of Microbiology, Dorevitch Pathology, 18 Banksia Street, Heidelberg, VIC 3084, Australia; Department of Infectious Diseases, The Alfred Hospital and Central Clinical School, Monash University, 85 Commercial Road, Melbourne, VIC 3004, Australia

## Abstract

**Background:**

Australian guidelines recommend trimethoprim or nitrofurantoin as first-line agents for uncomplicated urinary tract infections (UTIs). Laboratory surveillance indicates high rates of trimethoprim resistance among urinary bacterial isolates, but there are scant local clinical data about risk factors and impact of trimethoprim resistance.

**Objectives:**

To determine the prevalence, risk factors, mechanism and impact of resistance to first-line antibiotic therapy for uncomplicated UTIs in the community setting.

**Methods:**

A prospective observational study from October 2019 to November 2021 in four general practices in Melbourne, Australia. Female adult patients prescribed an antibiotic for suspected or confirmed uncomplicated acute cystitis were eligible. Primary outcome was urine isolates with resistance to trimethoprim and/or nitrofurantoin.

**Results:**

We recruited 87 participants across 102 UTI episodes with median (IQR) age of 63 (47–76) years. *Escherichia coli* was the most common uropathogen cultured (48/62; 77%); 27% (13/48) were resistant to trimethoprim (mediated by a *dfrA* gene) and none were resistant to nitrofurantoin. Isolates with resistance to a first-line therapy were more common among patients reporting a history of recurrent UTIs [risk ratio (RR): 2.08 (95% CI: 1.24–3.51)] and antibiotic use in the previous 6 months [RR: 1.89 (95% CI: 1.36–2.62)]. Uropathogen resistance to empirical therapy was not associated with worse clinical outcomes.

**Conclusions:**

Resistance to trimethoprim is common in uncomplicated UTIs in Australia but may not impact clinical outcomes. Further research is warranted on the appropriateness of trimethoprim as empirical therapy, particularly for patients with antimicrobial resistance risk factors.

## Introduction

Urinary tract infections (UTIs) are common in the community, accounting for 1.2% of all problems managed in Australian general practice.^1^ Uncomplicated acute cystitis is the most common UTI and most frequently caused by *Escherichia coli*, a member of the Enterobacterales order, which has an increasing prevalence of antimicrobial resistance (AMR).^2^ AMR increasingly complicates the management of UTIs and results in increasing morbidity, mortality and costs to the health system.^3–5^

There are global variations in treatment guidelines for uncomplicated acute cystitis and geographic variations of AMR in uropathogens. Trimethoprim (300 mg, daily, for 3 days) and nitrofurantoin (100 mg, 6 hourly, for 5 days) are the recommended empirical first-line therapies for uncomplicated acute cystitis in Australia.^6^ Australia is distinctive in recommending trimethoprim for cystitis, with guidelines in other countries usually favouring trimethoprim/sulfamethoxazole over trimethoprim alone.^7^ Australian AMR surveillance data indicate that 24% of urinary *E. coli* isolates are resistant to trimethoprim, but it’s unclear whether this represents a biased sample as patients without risk factors for resistance can be treated without collection of a midstream specimen of urine (MSU) for culture.^8^

There are scant data with varied results on the prevalence of resistance to antibiotic agents in uropathogens causing acute uncomplicated cystitis in general practice in Australia collected in conjunction with clinical risk factors and outcomes. Therefore, we aimed to determine the prevalence of resistance to first-line antibiotics used for the treatment of acute uncomplicated cystitis, describe whether resistance to first-line antibiotic therapy is predicted by pre-selected risk factors, and evaluate the impact of resistance to empirical therapy on clinical outcomes.

## Methods

### Ethics

This study was approved by Alfred Health Human Research Ethics Committee (HREC/19/52661).

### Study design, participants and setting

We performed a prospective cohort study of non-pregnant female adults (≥18 years old) prescribed an antibiotic for suspected or confirmed uncomplicated acute cystitis, as diagnosed by their GP, in primary care practices in Melbourne from October 2019 to November 2021. Non-English-speaking persons, patients with structural or functional abnormality or urinary tract device were excluded. For this primarily descriptive, hypothesis-generating study performed during the COVID-19 pandemic, the sample size was determined pragmatically by the study period.

### GP participation

We used several methods to recruit a convenience sample of GP practices; we sought expressions of interest at a Women’s Health symposium in Melbourne, via our research institution website, and via the Dorevitch Pathology GP newsletter. We also directly approached GP practices across Melbourne based on existing relationships, and through e-mail and phone calls to GP practices. All GP practices were offered an in-person presentation about the research study and UTI management.

### Participant recruitment

Participating GPs briefed potentially eligible patients about the study, provided a participant information sheet and, with permission, shared patient contact details and brief clinical information (see ‘Data collection’) to the research team using an online form. The research team called potential participants within 48 h of the GP consultation to obtain verbal informed consent for study participation. The research team had no influence on clinical management, including antibiotic selection and the decision to request an MSU. Australian guidelines recommend that an MSU be collected prior to empirical antibiotics for uncomplicated cystitis for patients with risk factors for AMR; however, there are scant data about the frequency with which this is requested in clinical practice.

### Data collection

Data collection for each participant occurred in four parts: GP survey; recruitment telephone survey by research team; microbiology results from MSU collection; and follow-up surveys via automated text messages. The GP survey included participant contact details, diagnostic tools used, and antibiotic prescription (agent, dose and duration). The recruitment telephone survey included demographics, UTI symptoms and duration, impact of the UTI on day-to-day wellbeing (on a scale of 0–10), antibiotic allergies and risk factors for AMR including: history of recurrent UTIs (self-reported history of two or more episodes in 6 months or three or more episodes in 12 months), residence in aged-care facility and use of antibiotics, international travel, or hospital admission in the past 6 months. Automated text messages were sent daily for the duration of the antimicrobial therapy or for a minimum of 4 days (whichever was longer) with a hyperlink to a survey to ask participants to select which symptoms they had, and whether they had attended a GP to seek further antibiotic treatment. An additional text message was sent 28 days following the GP consultation to assess symptoms and whether the patient had returned to a doctor for further antibiotics (yes, no). Data collection questions were restricted to focus on the study aims to minimize the burden of time for participation, and we did not explore further patient sequelae such as hospital presentation or admission. Microbiology results from Dorevitch Pathology were entered directly into the online database by the research team, and results from other pathology services were faxed or e-mailed to the research team for data entry. All data were collected using REDCap hosted on our institution’s server.^9,10^

### Isolate identification and phenotypic susceptibility testing

Isolate identification and phenotypic susceptibility testing were performed according to standard procedures at each pathology service. At Dorevitch Pathology, the MSU submitted for laboratory testing was cultured on CHROMID CPS Elite (CPSE) agar incubated overnight at 35°C–37°C in air (bioMérieux). Identification of isolates was confirmed using MALDI-TOF MS on the VITEK MS platform (bioMérieux). Susceptibility testing was performed according to CLSI guidelines using disc diffusion against ampicillin, amoxicillin/clavulanate, cefazolin, ceftriaxone, ciprofloxacin, trimethoprim and nitrofurantoin (Bio-Rad) and supplementary susceptibility testing to fosfomycin, meropenem, ertapenem and gentamicin was performed in the event of *E. coli* ceftriaxone non-susceptibility.^11^ Where susceptibility testing was not performed for *Staphylococcus saprophyticus*, we assumed trimethoprim susceptibility.^12^ We focused on *E. coli* for genomic characterization as it is the most frequently isolated bacterium in urine.

### WGS of E. coli isolates

Subcultures from each isolate identified as *E. coli* on the VITEK MS (bioMérieux) at Dorevitch Pathology were sent to an ISO-accredited laboratory for WGS. DNA extraction, Nextera XT library preparation, and sequencing on the Illumina NextSeq platform were performed as previously described.^13^ Bioinformatic analyses were undertaken using National Association of Testing Authorities accredited tools incorporated into a custom-built pipeline (https://github.com/MDU-PHL/bohra). Following quality control processing, short-read sequencing data for each isolate were assembled *de novo* by SPAdes (v3.15.2) to infer multi-locus sequence type using MLST (v2.19.0) (https://github.com/tseemann/mlst) with the PubMLST database (https://pubmlst.org/) and determine the presence of known AMR genes *in silico* with abriTAMR (v1.0.7) (https://github.com/MDU-PHL/abritamr). Additionally, the phylogenetic relationship of isolates was assessed through variant calling and core-genome alignment derived from read alignment to a reference genome (*E. coli* K-12 substr. MG1655)^14^ using Snippy (v4.4.5) (https://github.com/tseemann/snippy) and IQ-Tree (v2.1.4).

### Exposure and outcomes

The main outcome of interest was resistance to first-line antibiotic therapy for the treatment of uncomplicated cystitis based on the current Australian Therapeutic Guidelines, defined as resistance to trimethoprim and/or nitrofurantoin.^6^ We considered the following exposures to be potential risk factors for resistance to first-line antibiotics: recurrent UTIs, residence in aged-care facility and use of antibiotics, international travel, or hospital admission in the past 6 months. Potential risk factors were pre-selected from those reported in Australian guidelines.^6^ We examined the impact of resistance on empirical therapy through the following clinical outcomes: symptom resolution by the end of the antibiotic treatment course and at 28 days post antibiotic therapy initiation (urinary urgency, urinary frequency, dysuria, lower abdominal pain); return to GP for change in antibiotic treatment during their antibiotic treatment course and within 28 days of antibiotic therapy initiation. We also asked about new adverse symptoms following antibiotic therapy initiation (fever, rash, nausea, vomiting, diarrhoea).

### Data analysis

Descriptive analysis summarized covariates and estimated the prevalence of antibiotic use and resistance. Risk ratios were presented with 95% CI. All analyses and visualization were performed using R Version 4.0.2 (https://www.r-project.org/).

## Results

### Participants

**Figure 1.**
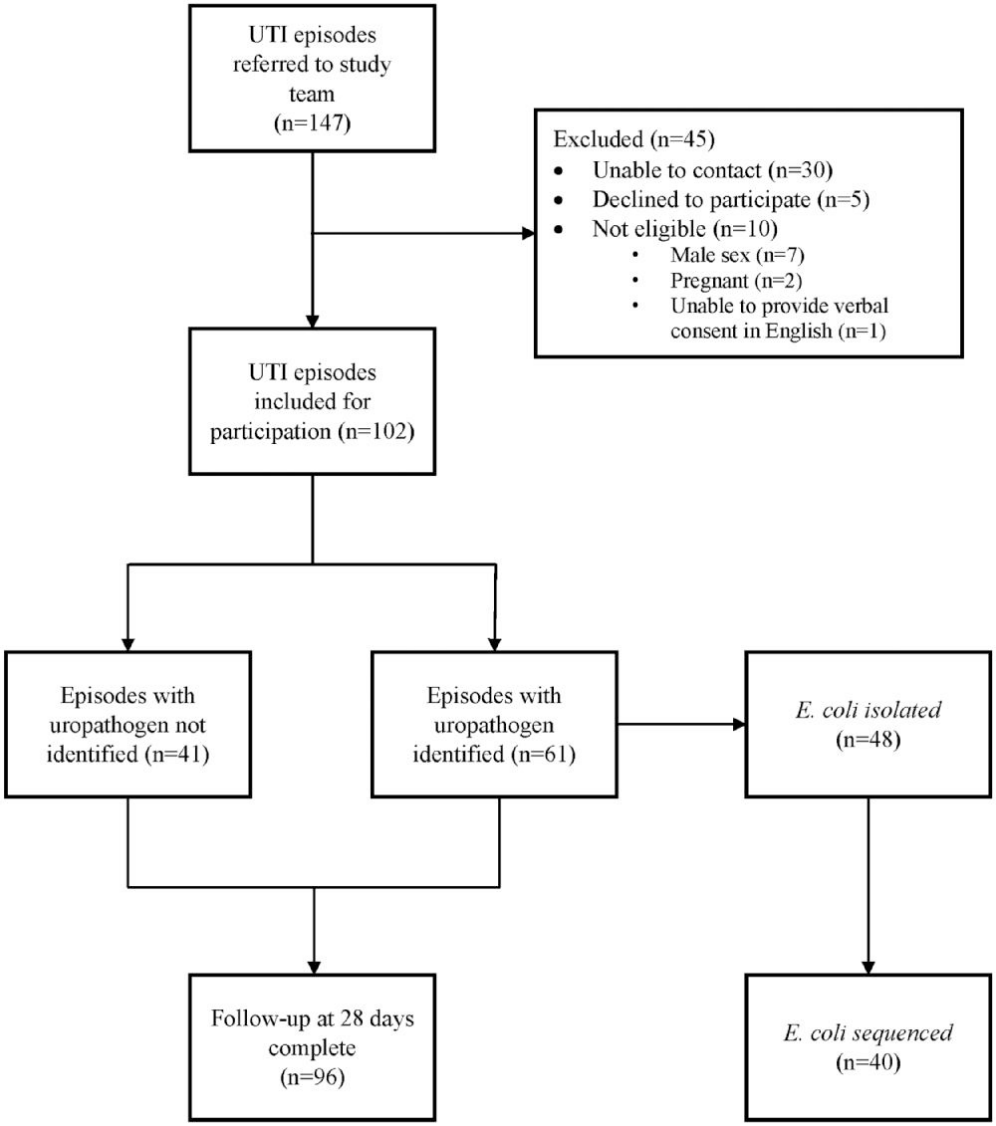
Flowchart of study participants, by UTI episode.

Of 137 potentially eligible patient presentations referred to the research team by four GP clinics across Melbourne, we recruited 102 (recruitment rate, 75%) involving 87 unique participants (Figure 1). The median age was 63 years (IQR: 47–76). Most participants had a urine dipstick performed (97/102; 95%) and all had an MSU requested for microscopy and culture, of which 88% (90/102) were sent to Dorevitch Pathology. Most participants (99/102; 97%) were symptomatic at GP consultation, and the median time from symptom onset to GP consultation was 3 days (IQR: 2–5). One-third (34/102) reported experiencing recurrent UTIs and the median time since previous UTI was 8 weeks (IQR: 4–20). Most participants were prescribed trimethoprim (77/102; 77%). Demographics, baseline characteristics and antibiotic therapy are presented in Table 1.

**Table 1.** Demographics and baseline characteristics of participants with uncomplicated acute cystitis in Melbourne, October 2019 to November 2021

Participant characteristic	*N* (%)
Total participants recruited at GP consultation	102
Total unique patients	87
Age (years), median (IQR)	63 (47–76)
Age group (years)	
<40	19 (19)
40–49	11 (11)
50–59	11 (11)
60–69	22 (21)
70–79	29 (28)
80+	10 (10)
Risk factors for AMR	
Antibiotic use in previous 6 months	51 (50)
Hospital admission in previous 6 months	35 (34)
Nights in hospital in previous 6 months, median (IQR)	4 (0–5)
International travel in previous 6 months	8 (8)
Residence in an aged-care facility	5 (5)
Recurrent UTI	34 (33)
Symptoms at GP consultation	99 (97)
Urinary frequency	74 (73)
Dysuria	53 (52)
Urinary urgency	46 (45)
Lower abdominal pain or pain above the pubic bone	39 (38)
Cloudy urine or change in colour of urine	24 (24)
New flank or back pain	17 (17)
Feeling feverish, shaking or chills	9 (8.8)
Impact of UTI on wellbeing, median (IQR)	6 (3–8)
Antibiotic allergy to trimethoprim or nitrofurantoin	4 (4)
Urinalysis	
Leucocyte esterase detected only	62 (61)
Nitrates detected only	13 (13)
Leucocyte esterase and nitrates detected	13 (13)
No abnormality detected	9 (9)
Not performed	5 (5)
Requested MSU	102 (100)
Pyuria (>40 × 10^6^/L WBCs)	77 (76)
Antibiotic agent prescribed (agent, dose, frequency, duration)	
Trimethoprim	77 (75)
300 mg, daily, 3 days	60 (59)
300 mg, daily, 5 days	5 (5)
300 mg, daily, 7 days	12 (12)
Nitrofurantoin, 100 mg, 6 hourly, 5 days	8 (8)
Cefalexin	8 (8)
500 mg, 6 hourly, 5 days	3 (3)
1 g, 12 hourly, 5 days	3 (3)
500 mg, 12 hourly, 5 days	2 (2)
Amoxicillin/clavulanate, 500 mg + 125 mg, 12 hourly, 5 days	6 (6)
Amoxicillin, 500 mg, 12 hourly, 5 days	2 (2)
Norfloxacin, 400 mg, 12 hourly, 7 days	1 (1)

### Bacterial identification and antimicrobial susceptibility

An organism was isolated from an MSU for 60% (61/102) of UTI episodes. *E. coli* (48/62 77%) was the most common, with smaller numbers of *S. saprophyticus* (5/62; 8.1%), *Klebsiella pneumoniae* (4/62; 7%), *Proteus mirabilis* (2/102; 3%), *Enterobacter aerogenes* (1/62; 2%), *Staphylococcus aureus* (1/62; 1.6%) and Group B *Streptococcus* (1/62; 2%). Half of isolates were resistant to ampicillin (29/56; 51.8%), and 26.5% (14/55) resistant to trimethoprim (Table 2).

**Table 2.** AMR in bacterial isolates from urine samples in participants with uncomplicated acute cystitis in Melbourne, October 2019 to November 2021

Antibiotic tested	All microorganisms			*E. coli*		
	Isolates resistant, *N*	Isolates tested, *N*	Resistance (%)	Isolates resistant, *N*	Isolates tested, *N*	Resistance (%)
Primary susceptibility testing						
Amoxicillin/clavulanate	5	55	9	4	48	8
Ampicillin	29	56	52	23	48	48
Cefazolin	8	55	15	7	48	15
Ciprofloxacin	9	51	18	9	44	21
Ceftriaxone	6	55	11	5	48	15
Nitrofurantoin	4	60	7	0	48	0
Trimethoprim	14	60	23	13	48	27
Supplementary susceptibility testing						
Ertapenem	0	7	0	0	6	0
Fosfomycin	0	6	0	0	6	0
Gentamicin	1	10	10	1	9	11
Meropenem	0	10	0	0	9	0
Trimethoprim/sulfamethoxazole	2	10	20	2	9	22

There were five (8.2%) ceftriaxone-resistant *E. coli* isolates, all of which had an ESBL phenotype. No isolate was meropenem resistant. Seven participants were prescribed trimethoprim but had a trimethoprim-resistant organism cultured. Table S1 (available as Supplementary data at *JAC-AMR* Online) presents participant antibiotic agent prescribed by trimethoprim susceptibility of bacterial isolates.

### Genomics

Of the 48 *E. coli* isolates identified, 40 underwent WGS. The isolates were derived from samples obtained from 35 individual participants: 32 with a single *E. coli* isolate, and three participants providing multiple *E. coli* isolates for sequencing. Twenty-four different STs were represented among the sequenced isolates. Phenotypic resistance to trimethoprim was evident in multiple *E. coli* STs and genomic lineages (Figure S1) and there was perfect correlation with the presence of a *dfrA* gene encoding a trimethoprim-insensitive dihydrofolate reductase. Among the sequenced isolates, *dfrA* was also frequently associated (8/10; 80%) with *bla*_TEM-1_ and phenotypic resistance to ampicillin, as well as genotypic and phenotypic resistance to sulphonamides (9/10; 90%).

### Predictors of resistance to first-line antibiotics

A total 68% (69/102) of participants had one or more risk factors for AMR. Participants with an organism isolated that was resistant to one or more first-line agents (trimethoprim or nitrofurantoin) more commonly reported one or more risk factors for AMR than those without resistance to first-line agents: 88% (15/17) versus 64% (54/85), respectively; risk ratio: 1.39 (95% CI: 1.10–1.76, *P* = 0.048). The positive and negative predictive values of having any risk factor for resistance were 88% (15/17) and 37% (31/85), respectively. The association between each risk factor and resistance to first-line antibiotics are reported in Table 3.

**Table 3.** Participant risk factors of resistance to first-line antibiotic therapy with uncomplicated acute cystitis in Melbourne, October 2019 to November 2021

	Organism resistant to a first-line agent, % (*N*)	No organism, organism susceptible to first-line agents or susceptibility testing not performed, % (*N*)	Risk ratio (95% CI)	*P* value
Recurrent UTI	59 (10/17)	28 (24/85)	2.08 (1.24–3.51)	0.015
Antibiotic use in previous 6 months	82 (14/17)	44 (37/85)	1.89 (1.36–2.62)	0.003
Hospital admission in previous 6 months	53 (9/17)	31 (26/85)	1.73 (0.99–3.00)	0.08
International travel in previous 6 months	6 (1/17)	8 (7/85)	0.71 (0.10–5.44)	0.74
Residence in an aged-care facility	6 (1/17)	5 (4/85)	1.25 (0.15–10.50)	0.84

### Patient outcomes

Overall, 67% (61/91) of participants reported symptom resolution by the end of their antibiotic treatment course, while 28% (27/96) remained symptomatic at 28 days post antibiotic therapy initiation. Twenty-nine of 96 participants (30%) returned to the GP for further treatment within 28 days of antibiotic therapy initiation, of which 18% (17/97) had returned during their antibiotic treatment course. There were no differences in clinical outcomes between participants with a uropathogen isolated compared with those without a uropathogen isolated, nor between participants with a uropathogen resistant to the prescribed antibiotic therapy (Table S2). Additionally, 11% (11/97) experienced new symptoms following antibiotic initiation; eight (8%) had nausea, vomiting or diarrhoea, two (2%) had both rash and fever, shaking or chills and one (1%) had nausea, vomiting or diarrhoea and fever, shaking or chills.

## Discussion

We found that a quarter of uropathogens causing uncomplicated cystitis among women were resistant to the first-line antibiotic therapy trimethoprim, while resistance to nitrofurantoin was rare. Trimethoprim resistance was more common among participants with recurrent UTIs, antibiotic use in the previous 6 months and hospital admission in the previous 6 months. Together, asking about these risk factors had relatively high positive predictive value for AMR. We were unable to demonstrate that either non-isolation of a uropathogen, or prescription of empirical therapy to which the isolate was resistant, resulted in worse outcomes.

We report estimates of *E. coli* resistance in urine isolates that were similar to Australian laboratory-based surveillance data; 27% versus 24% for trimethoprim, and 0% versus 1% for nitrofurantoin, respectively.^8^ We also report similar rates of *E. coli* with ESBL phenotypes in urine Australian laboratory-based surveillance data: 8% versus 7%.^14^ Laboratory-based surveillance could potentially overestimate the prevalence of AMR in community uropathogens, as national guidelines only recommend collection of an MSU for patients with risk factors. In our study, however, over two-thirds of patients had one or more risk factors for resistance and all patients had an MSU.^6^ This may support that national laboratory-based surveillance of AMR among urinary isolates can be representative of the community setting.

In our cohort, no pathogen was identified from MSU samples in one-third of patients. Although lower urinary tract symptoms are most frequently due to bacterial infection, there may be non-bacterial causes. Unlike other studies that enrolled patients once a uropathogen was isolated, in our study, patients were enrolled on clinical suspicion of a UTI, which may explain the relatively high rate of patients with culture-negative urine specimens.^15–18^ Additionally, in this study, urine specimens were cultured onto chromogenic CPSE agar with an overnight incubation only, and standard urine culture may fail to detect slow-growing, fastidious, and non-aerobic organisms such as *Aerococcus* species and *Actinotignum schaalii*, which may explain culture-negative patients with persistent symptoms returning to a GP.^4,19,20^ However, because multidrug resistance has largely been documented in Enterobacterales, which are non-fastidious and rapid growers, we do not anticipate that this will have significantly affected our estimates of antibiotic resistance.

Trimethoprim resistance was more common in patients who reported antibiotic use in the preceding 6 months and in those with recurrent UTIs than other patients. Trimethoprim resistance was not due to prominence of a dominant uropathogenic clonal type but was rather seen across multiple STs and genetic lineages. The presence of risk factors in patients with trimethoprim-resistant uropathogens highlights the need for MSUs to be submitted for laboratory testing in patients with risk factors for AMR, or for consideration of alternative empirical therapy to prevent clinical failure. Whilst trimethoprim resistance in uropathogens is increasing globally, nitrofurantoin resistance remains consistently lower, which may make nitrofurantoin the preferable empirical therapy for patients with risk factors.^21–23^ However, the dosing regimen for trimethoprim (daily, for 3 days) is more convenient for patients than nitrofurantoin (four times daily, for 5 days). The slow-release twice-daily dosing of nitrofurantoin (Macrobid^®^, Procter and Gamble Pharmaceuticals, Inc.) is not currently available in Australia. Furthermore, utilization of nitrofurantoin may not be suitable for all populations due to disease and pharmaceutical interactions, which may make trimethoprim favourable in these populations.

Clinical failure was common in our cohort; however, uropathogen resistance to empirical therapy was not associated with worse clinical outcomes, nor was culture positivity of an MSU. There is varied evidence on clinical outcomes of patients with uncomplicated acute cystitis, with no standardized reporting of endpoints. In six GP clinics in Ireland, 25% of patients returned to a GP for further treatment by Day 28, in 10 GP clinics across England, Netherlands, Spain and Wales, 24% of patients had consulted a GP for persistent symptoms and 10% were prescribed a subsequent antibiotic at 2 weeks post-antibiotic initiation.^24,25^ The clinical outcomes of our cohort were similar to these estimates; however, we did not see similar outcomes to previous literature that reports poorer clinical outcomes according to presence of uropathogen resistance.^15–18,24–26^ Symptom severity and persistence in patients with uropathogen resistance, compared with patients with uropathogen susceptibility, was seen in 67 primary care practices in England and in 10 general practices in South Wales, regardless of whether the patient was treated with an antibiotic to which the uropathogen was susceptible.^15,26^ Conversely, in 59 general practices in Denmark, there was no significant difference in symptom severity or duration for patients with a resistant or susceptible *E. coli* uropathogen if patients were treated with an antibiotic to which the uropathogen was susceptible.^18^ The apparent efficacy of trimethoprim to resolve symptoms in our patients infected with trimethoprim-resistant isolates may be due to high concentrations achieved in the urinary tract with urinary concentrations of about 100 μg/mL after the usual oral dose.^27^ In addition, there is evidence that uncomplicated UTIs spontaneously resolve or can be treated by non-antibiotic treatments, although use of non-antibiotic treatments such as non-steroidal anti-inflammatory drugs was not captured in our study.^4,28^ Given the modest number of participants in our study, this finding is hypothesis-generating and should be examined further.

Our study has some limitations. First, while patients were recruited from four geographically distinct GP clinics in Melbourne, most patients were recruited from one GP practice in the east of Melbourne, and we excluded patients unable to perform verbal consent in English. This, along with the use of a convenience sample and a cohort of an older average age than patients who present to GPs for uncomplicated cystitis, may impact on the generalizability of our findings because there is sociodemographic and geographic variation in uropathogen resistance.^29^ Second, participation bias may be caused if patients with recurrent UTIs were more willing to participate than those experiencing infrequent UTIs; however, the participating GP facilitated the identification of a range of potential participants. Third, some participant symptoms at enrolment deviated from standard diagnostic criteria for uncomplicated UTIs, including three participants who denied symptoms when questioned by the research team. This may be attributed to limitations of self-report data, and ultimately, patients were enrolled based on antibiotics prescribed for an indication of a UTI by their treating GP. Fourth, there was variation in participant follow-up beyond baseline resulting in missing outcome data; however, 94% of participants responded at the 28 day follow-up. Non-participation during follow-up may have occurred due to being unwell, inconvenience of texting or lack of interest. Fifth, our sample size was smaller than anticipated and we were unable to perform multivariable analysis; however, overall, we have contributed to the currently scant availability of clinical data about risk factors and impact of AMR in uncomplicated UTIs in the Australian community setting. Further larger studies can contribute to this body of knowledge to inform national guidelines, including identification of optimal management strategies for patients with uncomplicated UTIs, including use of MSU and empirical therapy.

### Conclusions

We found that resistance to first-line therapy for acute uncomplicated cystitis was common in Australia, supporting the accuracy of laboratory-based surveillance. We did not, however, find that resistance impacted on clinical outcomes. Our study contributes to an understanding of local AMR patterns in the community in Melbourne and further research is warranted on the role of trimethoprim as empirical therapy and the impact of uropathogen resistance on clinical outcomes among patients with uncomplicated cystitis.

## Supplementary Material

dlad145_Supplementary_Data

## Data Availability

The data that support the findings of this study are available from the corresponding author, S.J.C., upon reasonable request, subject to approval by the relevant institutional review boards.

## Supplementary data

Figure S1 and Tables S1 and S2 are available as Supplementary data at *JAC-AMR* Online.
