# Supplementary material for: Resistance to first-line antibiotic therapy among patients with uncomplicated acute cystitis in Melbourne, Australia: prevalence, predictors and clinical impact
Source: JAC Antimicrob Resist. 2023 Dec 28;6(1):dlad145. doi: 10.1093/jacamr/dlad145 (PMC10753919; doi:10.1093/jacamr/dlad145)
Supplement: dlad145_Supplementary_Data [file dlad145_supplementary_data.docx]

**Table S1. Antibiotic agent prescribed to participants and participant uropathogen resistance, by trimethoprim and prescribed antibiotic agent in women with uncomplicated acute cystitis in Melbourne, October 2019 to November 2021.**

| **Antibiotic agent prescribed** | **Participant prescribed agent,**  **N** | **Susceptibility to trimethoprim** | | | **Susceptibility to prescribed antibiotic agent** | | |
| --- | --- | --- | --- | --- | --- | --- | --- |
|  |  | **No organism or susceptibility testing not performed,**  **N** | **Trimethoprim susceptible organism,**  **N** | **Trimethoprim resistant organism,**  **N** | **No organism or antibiotic susceptibility testing not performed, N** | **Susceptible organism,**  **N** | **Resistant organism,**  **N** |
| Trimethoprim | 77 | 30 | 40* | 7 | 30 | 40* | 7 |
| Nitrofurantoin | 8 | 5 | 3 | 0 | 5 | 3 | 0 |
| Cephalexin | 8 | 4 | 1 | 3 | 4 | 3 | 1 |
| Amoxicillin-clavulanate | 6 | 1 | 1 | 4 | 1 | 4 | 1 |
| Amoxicillin | 2 | 2 | 0 | 0 | 2 | 0 | 0 |
| Norfloxacin | 1 | 1 | 0 | 0 | 1 | 0 | 0 |
| **One participant prescribed trimethoprim had two trimethoprim susceptible bacterial isolates; included here once.* | | | | | | | |

**Figure S1. Phylogenetic tree with predicted antibiotic resistance of *Escherichia coli* isolates**

Maximum likelihood phylogenetic tree based on core genome pairwise SNP profiles. Heatmap rows summarising the results of antimicrobial susceptibility assays are aligned to the corresponding isolate on the tree tip.

**Table S2. Clinical outcomes of participants with uncomplicated acute cystitis in Melbourne by uropathogens sensitivity, October 2019 to November 2021.**

|  | **Uropathogen resistant to prescribed antibiotic,**  N (%) | **Uropathogen susceptible to prescribed antibiotic,**  N (%) | **Risk Ratio [95%CI]** | **P-value** |
| --- | --- | --- | --- | --- |
| Return to GP for change in antibiotic treatment during their antibiotic treatment course | 1/9 (11) | 8/49 (16) | 0.68 [0.10, 4.80] | 0.69 |
| Symptom resolution by end of antibiotic treatment course | 5/8 (63) | 34/46 (74) | 0.85 [0.48, 1.49] | 0.51 |
| Symptomatic at 28 days post antibiotic therapy initiation | 3/9 (33) | 15/49 (31) | 1.09 [0.40, 3.01] | 0.87 |
| Return to GP for further treatment within 28 days of antibiotic therapy initiation | 3/9 (33) | 15/49 (31) | 1.09 [0.40, 3.01] | 0.87 |
